# Risk factor analysis for postoperative complications requiring revision surgery after transtrochanteric rotational osteotomy for osteonecrosis of the femoral head

**DOI:** 10.1186/s13018-018-0714-4

**Published:** 2018-01-10

**Authors:** Kazuyuki Karasuyama, Goro Motomura, Satoshi Ikemura, Jun-ichi Fukushi, Satoshi Hamai, Kazuhiko Sonoda, Yusuke Kubo, Takuaki Yamamoto, Yasuharu Nakashima

**Affiliations:** 1 Department of Orthopaedic Surgery, Graduate School of Medical Sciences, Kyushu University, 3-1-1 Maidashi, Higashi-ku, Fukuoka, Japan; 2 Department of Orthopaedic Surgery, Faculty of Medicine, Fukuoka University, 7-45-1 Nanakuma, 12 Jonan-ku, Fukuoka, Japan

**Keywords:** Alcohol abuse, Corticosteroid, Transtrochanteric rotational osteotomy, Osteonecrosis of the femoral head

## Abstract

**Background:**

This study investigated the risk factors for postoperative complications requiring revision surgery within 3 years after transtrochanteric rotational osteotomy (TRO) for osteonecrosis of the femoral head (ONFH).

**Methods:**

We reviewed 127 patients (147 hips) who underwent TRO (anterior or posterior rotational osteotomy) for ONFH between January 2002 and December 2014. Two patients were lost to follow-up, and five patients with progression of femoral head collapse requiring a salvage procedure such as total hip arthroplasty within 3 years after TRO were excluded. The better hip in patients treated bilaterally was also excluded (*n* = 20) to avoid duplication of patient demographics, leaving 120 hips (120 patients) for the analysis. We reviewed the medical records of each patient to screen for postoperative complications that required revision surgery within 3 years after surgery, recording the patient’s age, sex, body mass index, surgical side, condition of the contralateral hip, previous alcohol intake, previous alcohol abuse, previous corticosteroid use, perioperative corticosteroid use, smoking status, preoperative stage and type of ONFH, preoperative activity level, and preoperative and final follow-up Japanese Orthopaedic Association scores. Differences between cases with and without complications were analyzed.

**Results:**

Eleven (9.2%) cases showed postoperative complications that required revision surgery. The most common complication was deep infection (*n* = 5), followed by nonunion of the greater trochanter (*n* = 3), nonunion of the intertrochanteric osteotomy site (*n* = 2), and femoral head fracture (*n* = 1). The multivariate analysis showed an independent association between previous alcohol abuse and postoperative complications (odds ratio, 13.5).

**Conclusion:**

A correlation might exist between alcohol abuse and complications following a TRO procedure.

## Background

Osteonecrosis of the femoral head (ONFH) often causes its collapse, leading to secondary osteoarthritis of the hip joint [1, 2]. Although the precise pathophysiological mechanisms underlying the development of ONFH remain unclear, both alcohol abuse and corticosteroid use have been shown to be associated with ONFH [3–5]. Because ONFH often occurs in younger adults, several procedures that preserve the femoral head have been developed to avoid or delay the need for joint replacement surgery [6–9].

In 1978, Sugioka proposed transtrochanteric rotational osteotomy (TRO) as a joint-preserving procedure for the treatment of ONFH [6]. This procedure leads to excellent clinical outcomes if patients are appropriately selected, the procedure is accurately performed, and appropriate postoperative rehabilitation is provided [7–13]. However, postoperative complications that require revision surgery after TRO may result in poor clinical outcomes. Indeed, in their later work, Sugioka and colleagues [13] reported the incidence of several postoperative complications after anterior rotational osteotomy, including deep infection, nonunion of the intertrochanteric osteotomy site, femoral neck fracture, and fracture of the lesser trochanter. Similarly, Saito and coworkers [14] reported postoperative complications such as femoral neck fracture and late varus deformity in 5 (33%) of 15 cases treated by the same method. However, no previous studies have reported the factors affecting postoperative complications after TRO for ONFH that require revision surgery. Thus, the purpose of this study was to investigate the risk factors for postoperative complications requiring revision surgery within 3 years after TRO for ONFH.

## Methods

### Patients

Our institutional review board approved this retrospective study. TRO for ONFH was performed on 147 hips of 127 patients at our institution between January 2002 and December 2014. Two patients were followed up for less than 3 years, and five patients with progression of femoral head collapse requiring a salvage procedure such as total hip arthroplasty within 3 years after TRO were excluded from this study. For the 20 patients who underwent bilateral TRO, only one hip was included in this study to avoid duplication of background characteristics in the analyses. The hip selected for inclusion in the study was determined according to the following inclusion parameters: (1) where postoperative complications occurred in both hips and one side required surgical treatment, the side requiring surgery was included; (2) where postoperative complications occurred in both hips and no revision surgery was required, the side with the lower (more severe) preoperative Japanese Orthopaedic Association (JOA) score was included; (3) where postoperative complications occurred in only one hip, the side with the complications was included; and (4) where there were no complications in either hip, the side with the lower preoperative JOA score was included. The JOA score consists of 40 points for pain, 20 points for a range of motion, 20 points for walking ability, and 20 points for the activity of daily living, with a maximum total score of 100 points [15].

This study therefore included 120 hips from 120 cases. All cases were followed up for more than 3 years after surgery. The cases were 91 males and 29 females with a mean age of 40 years (range 15–60 years) at the time of surgery. The mean follow-up period was 8.9 years (range 3.0–15.7 years).

The age, sex, body mass index, surgical side, condition of the contralateral hip, previous alcohol intake, previous alcohol abuse, previous corticosteroid use, perioperative corticosteroid use, smoking status, preoperative stage and type of ONFH, preoperative activity level, and preoperative and final follow-up JOA scores were recorded for each patient.

Previous alcohol intake was defined as the ingestion of ≥ 400 ml of pure ethanol per week (40 standard drinks) [16, 17], whereas previous alcohol abuse was defined as the ingestion of ≥ 1000 ml of pure ethanol per week (100 standard drinks per week) [16, 17]. The preoperative activity level was classified as high, moderate, or low, indicating heavy work, standing work, or desk work, respectively [16]. Previous corticosteroid use was defined as previous oral or transvenous corticosteroid therapy. Perioperative corticosteroid use was defined as perioperative oral or transvenous corticosteroid therapy. In this study, the use of corticosteroids during the perioperative period was not standard therapy. However, the continuous administration of corticosteroids was required in 38 patients during the perioperative period because of systemic inflammatory conditions such as collagen diseases, including systemic lupus erythematosus.

The degree of ONFH was classified according to the guidelines set by the Japanese Investigation Committee of the Ministry of Health, Labour and Welfare [18]. Eighty-seven cases were classified as stage 3A, indicating collapse of the femoral head of < 3 mm; 32 cases were classified as stage 3B, indicating collapse of the femoral head of ≥ 3 mm; and one case was classified as stage 4, indicating osteoarthritic change.

Fifty patients had previous alcohol intake; 38 had a history of corticosteroid use, including 21 patients who received continuous corticosteroid treatment during the perioperative period; 28 patients had a history of alcohol intake plus corticosteroid use, including 17 who received continuous corticosteroid treatment during the perioperative period; and 4 patients were idiopathic.

The mean prednisolone-equivalent corticosteroid dose during the perioperative period was 6.5 mg/day (range 2–10 mg/day) in patients without previous alcohol intake and 6.3 mg/day (range 1–15 mg/day) in cases with previous alcohol intake.

### Operative and postoperative protocols

Anterior rotational osteotomy was performed when necrosis was localized to the anterior portion of the femoral head [6], and posterior rotational osteotomy was performed when there was necrosis in the middle and posterior portions of the femoral head [7, 8]. The surgical technique used was essentially the same as the originally described technique [6]. Three osteotomies were performed: (1) an osteotomy of the greater trochanter, (2) an intertrochanteric osteotomy from the superolateral to the inferomedial aspects on an anteroposterior view, and (3) an osteotomy from the proximal flare of the lesser trochanter, inferomedially towards the inferomedial extent of the primary osteotomy [19]. After anterior or posterior rotation of the proximal fragment, an 8.5-mm-diameter lag screw was inserted into the femoral head, and a modular-type plate was placed on the lateral side of the femur using one or two large cancellous screws [20]. The greater trochanter was re-attached using a heaving bailing wire [6].

In general, patients were instructed to remain non-weight bearing for 5 weeks, after which partial weight bearing was strictly performed for 3 weeks. Thereafter, walking with a stick was initiated until the bone union was confirmed. Full weight bearing was usually permitted after 6 months. If nonunion of the intertrochanteric osteotomy site, including varus deformity, was suspected, non-weight bearing or partial weight bearing was required until the bone union was confirmed.

### Assessment of postoperative complications

Postoperative complications requiring revision surgery occurring within 3 years after TRO were identified by reviewing the medical records and serial radiographs. Nonunion was defined as incomplete bone healing at 6 months after surgery. Three of the authors (KK, KS, and YK), who were not involved in surgical management, reviewed the radiographs to identify nonunion of the osteotomy site and the greater trochanter. Deep infection was defined as an infection occurring beneath the incision site in the muscle and in the tissue surrounding the muscle, which was confirmed using magnetic resonance imaging [21].

### Statistical analysis

Age, body mass index, and preoperative and final follow-up JOA scores were compared between cases with and without postoperative complications using an unpaired *t* test. Sex, surgical side, condition of the contralateral hip, previous alcohol intake, previous alcohol abuse, previous corticosteroid use, perioperative corticosteroid use, smoking status, preoperative stage and type, and preoperative activity level were compared between cases with and without complications using Fisher’s exact probability test.

Multivariate logistic regression analysis was performed to identify the preoperative factors associated with postoperative complications requiring revision surgery. Variables with a *p* value of < 0.2 in the univariate analyses (without the final follow-up JOA score) were included in the multivariate analysis. All statistical analyses were performed using JMP 11.0 software (SAS Institute, Cary, NC, USA). Values of *p* < 0.05 were considered statistically significant.

## Results

**Fig. 1:**
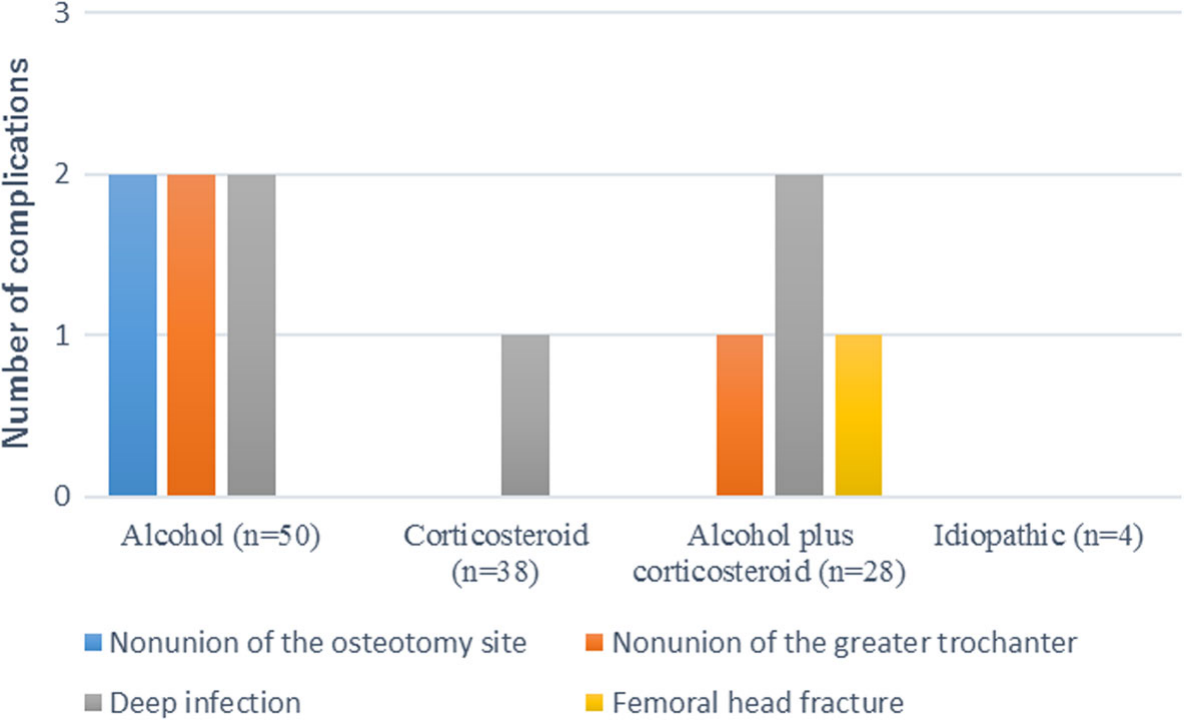
Number of postoperative complications requiring revision surgery within 3 years after transtrochanteric rotational osteotomy, according to previous alcohol intake and corticosteroid use. The postoperative complications were observed in six patients with alcohol, followed by four patients with alcohol plus corticosteroid, and one patient with corticosteroid. No complication was observed in patients with idiopathic

Among the 120 patients, 11 postoperative complications required revision surgery (9.2%). The most common complication requiring revision surgery was deep infection (*n* = 5), followed by nonunion of the greater trochanter (*n* = 3), nonunion of the intertrochanteric osteotomy site (*n* = 2), and femoral head fracture (*n* = 1) (Fig. 1). Five cases of deep infection were treated with surgical debridement and irrigation. Three cases of nonunion of the greater trochanter and one case of nonunion of the intertrochanteric osteotomy site were treated with reduction and internal fixation using the screw and wire approach. One case of nonunion of the intertrochanteric osteotomy site required bipolar hip arthroplasty with bone grafting. Osteosynthesis was performed in one case of femoral head fracture. Vessel injury, hematoma, and sciatic nerve palsy were not observed. The mean period from the initial surgery to the revision surgery was 4.3 months (range 0.6–16.5 months).

Among the 50 patients with previous alcohol intake but no corticosteroid use, 6 (12.0%) patients had postoperative complications that required revision. In contrast, among the 38 cases with previous corticosteroid use but no alcohol intake, only 1 (2.6%) postoperative complication requiring revision surgery was observed. Among the 28 cases with previous alcohol intake plus corticosteroid use, 4 (14.3%) required postoperative revision (Fig. 1). The overall mean preoperative JOA score was 56.5 (range 25–87), which improved to 84.9 (range 50–100) at the final follow-up.

Univariate analyses showed significant differences between cases with and without postoperative complications in terms of previous alcohol abuse (*p* = 0.0001; Table 1) and final follow-up JOA score (*p* = 0.0169; Table 1). The final follow-up JOA score was significantly lower in cases with complications than in cases without complications. There were no significant differences in age, sex, body mass index, surgical side, condition of the contralateral hip, previous alcohol intake, previous corticosteroid use, perioperative corticosteroid use, preoperative stage and type, preoperative activity level, or preoperative JOA score between cases with and without complications. Previous alcohol abuse (not previous alcohol intake) was included in the multivariate analysis to exclude the influence of confounding factors. The multivariate analysis showed an independent association between previous alcohol abuse and postoperative complications (odds ratio 13.5) (Table 2).

**Table 1:** Univariate analyses of factors associated with postoperative complications requiring revision surgery

	Complications (*n* = 11)	No complications (*n* = 109)	*p* value
Sex			
Male	8	83	0.8007
Female	3	26	
Age (years), mean (range)	41.4 (25–53)	39.6 (15–60)	0.6500
Body mass index (kg/m^2^)	23.8 (17.6–29.1)	22.9 (15.6–34.8)	0.2767
Surgical side			
Right	6	54	0.7517
Left	5	55	
Contralateral hip			
ON (+)	9	62	0.1088
ON (−)	2	47	
Activity level			
High	1	17	0.7274
Moderate	4	45	
Low	6	47	
Previous alcohol intake			
Yes	10	68	0.0587
No	1	41	
Previous alcohol abuse			
Yes	8	20	0.0001*
No	3	89	
Previous corticosteroid use			
Yes	5	61	0.5043
No	6	48	
Perioperative corticosteroid use			
Yes	2	36	0.3131
No	9	73	
Smoking			
Yes	8	70	0.6130
No	3	39	
Type			
C-1	3	38	0.6130
C-2	8	71	
Stage			
3A	7	80	0.7192
3B	4	28	
4	0	1	
Preoperative JOA score	58.40 (44–73)	57.22 (25–84)	0.5894
Final follow-up JOA score	79.10 (70–86)	85.56 (52–100)	0.0169*

*ON* osteonecrosis; *JOA* Japanese Orthopaedic Association

*p < 0.05 considered significant

**Table 2:** Multivariate logistic regression analysis for factors associated with postoperative complications requiring revision surgery

	Odds ratio (95% CI)	*p* value
Previous alcohol abuse	13.54 (3.16–57.98)	0.0004*
Contralateral hip	4.38 (0.82–23.49)	0.0571

*CI* confidence interval

*p < 0.05 considered significant

## Discussion

In this study, previous alcohol abuse was found to be an independent risk factor for revision surgery within 3 years after TRO. We found no relationship between previous or perioperative corticosteroid use and the risk of postoperative complications.

Regarding postoperative complications after TRO, Sugioka and colleagues [6] reported three hips with early complications (3/474), including one hip with lesser trochanter fracture and two hips with deep infection, followed by nine hips with late complications (9/474), including four hips with femoral neck fracture and five hips with delayed union of the osteotomy site after TRO. However, a high rate of postoperative complications after TRO was observed. Saito and coworkers [14] reported postoperative complications, including three hips with a femoral neck fracture and two hips with late varus deformity, in 5 (33%) of 15 hips, which subsequently required salvage procedures such as prosthetic replacement. In our study, the most common complication was five cases of deep infection, followed by three cases of nonunion of the greater trochanter, two cases of nonunion of the intertrochanteric osteotomy site, and one case of femoral head fracture. The complication rates in these three studies were 2.5% (12/474; Sugioka [6]), 33% (5/15; Saito et al. [14]), and 9.2% (11/120; this study). The complication rate of this study was higher than that of Sugioka and markedly lower than that of Saito et al.

Nonunion of the intertrochanteric osteotomy site or the greater trochanter accounted for 45.5% (5/11) of the postoperative complications observed in this study. Duckworth and colleagues [22] reported that alcohol abuse was the strongest predictor of postoperative complications after fixation of a displaced femoral neck fracture, with nonunion being the most common complication. Some previous studies found that alcohol may have a direct negative effect on bone healing via inhibition of osteoblast function [23, 24]. In a previous animal study, rats that underwent osteotomy of both femora after 1 year of alcohol consumption had a total absence of callus, whereas the femora in alcohol-free controls had fully healed, with a mineralized callus [25]. As might be expected, dose-dependent effects of ethanol on bone repair in rats have also been reported, with higher doses of ethanol resulting in deficiencies in bone repair as compared with lower doses [26]. These findings suggest that nonunion of an osteotomy site or the greater trochanter after TRO may be associated with previous alcohol abuse. In our study, with consideration of the dose-dependent effects of ethanol, alcohol consumption was divided into ingestion of ≥ 400 or ≥ 1000 ml of pure ethanol per week.

Deep infection accounted for 45.5% (5/11) of the postoperative complications observed in this study. Cordero-Ampuero and others [27] reported that alcohol abuse is a risk factor for infection after hemiarthroplasty or total hip arthroplasty. Alcohol abuse is also a significant risk factor for wound infection after spinal surgery [28]. Although corticosteroid use is associated with postoperative infection [29, 30], we did not find an association between previous or perioperative corticosteroid use and postoperative complications after TRO. Of the 38 cases with previous corticosteroid use, only 21 received continuous corticosteroid treatment during the perioperative period. A study of surgical site infection after the repair of mandibular fractures found that perioperative high-dose corticosteroid therapy increased the risk of postoperative infection [30]. The lack of association between previous or perioperative corticosteroid use and postoperative complications in this study may be related to the relatively low corticosteroid dose (prednisolone-equivalent dose of 6.5 mg/day in the 21 cases who received continuous corticosteroid treatment) and the relatively low proportion of cases who received perioperative corticosteroid treatment.

Postoperative complications requiring revision surgery are important factors influencing postoperative clinical outcomes. In this study, the final follow-up JOA score was lower in cases with complications than in cases without complications. To our knowledge, previous alcohol intake (ingestion of ≥ 400 ml of pure ethanol) has not previously been reported to affect outcomes after TRO. Zhao and colleagues [11] found that outcomes after anterior rotational osteotomy were associated with the postoperative intact ratio but not with etiology. Although it is important to recognize that patients with previous alcohol abuse (ingestion of ≥ 1000 ml of pure ethanol) have an increased risk of revision surgery after TRO, the indications for surgery should not be excessively limited by this factor. TRO should still be performed in patients with previous alcohol abuse, with the appropriate patient selection, accurate surgical technique, and appropriate postoperative rehabilitation.

This study has several limitations. First, the TRO procedures were not performed by a single surgeon. However, all surgeons who performed TRO were specialized in hip joint surgery. As the operating surgeon or the supervisor was always an experienced surgeon, we consider that the particular surgeon who performed the procedure was highly unlikely to have substantially affected the outcome. Second, differences in muscular strength or reported pain between the patients might have affected the progress of rehabilitation. However, it is unclear whether differences in the degree of progress between patients might affect the occurrence of complications. Finally, the dose of previous corticosteroid use could not be clearly determined upon review of the medical records, and it is possible that the relationship between current and previous doses of corticosteroids might have influenced the occurrence of postoperative complications.

## Conclusion

A correlation might exist between alcohol abuse and complications following a TRO procedure.

## Abbreviations

JOA: Japanese Orthopaedic Association
ONFH: Osteonecrosis of the femoral head
TRO: Transtrochanteric rotational osteotomy

## References

[1] Lieberman JR, Berry DJ, Mont MA, Aaron RK, Callaghan JJ, Rajadhyaksha AD (2003). Osteonecrosis of the hip: management in the 21st century. Instr Course Lect.

[2] Mankin HJ (1992). Nontraumatic necrosis of bone (osteonecrosis). N Engl J Med.

[3] Hirota Y, Hirohata T, Fukuda K, Mori M, Yanagawa H, Ohno Y (1993). Association of alcohol intake, cigarette smoking, and occupational status with the risk of idiopathic osteonecrosis of the femoral head. Am J Epidemiol.

[4] Fukushima W, Fujioka M, Kubo T, Tamakoshi A, Nagai M, Hirota Y (2010). Nationwide epidemiologic survey of idiopathic osteonecrosis of the femoral head. Clin Orthop Relate Res.

[5] Sakaguchi M, Tanaka T, Fukushima W, Kubo T, Hirota Y (2010). Idiopathic ONF Multicenter Case-Control Study Group: impact of oral corticosteroid use for idiopathic osteonecrosis of the femoral head: a nationwide multicenter case-control study in Japan. J Orthop Sci.

[6] Sugioka Y (1978). Transtrochanteric anterior rotational osteotomy of the femoral head in the treatment of osteonecrosis affecting the hip: a new osteotomy operation. Clin Orthop Relate Res.

[7] Atsumi T, Muraki M, Yoshikawa S, Kajihara T (1999). Posterior rotational osteotomy for the treatment of femoral head osteonecrosis. Arch Orthop Trauma Surg.

[8] Sugioka Y, Yamamoto T (2008). Transtrochanteric posterior rotational osteotomy for osteonecrosis. Clin Orthop Relate Res.

[9] Motomura G, Yamamoto T, Suenaga K, Nakashima Y, Mawatari T, Ikemura S (2010). Long-term outcome of transtrochanteric anterior rotational osteotomy for osteonecrosis of the femoral head in patients with systemic lupus erythematosus. Lupus.

[10] Miyanishi K, Noguchi Y, Yamamoto T, Irisa T, Suenaga E, Jigushi S (2000). Prediction of the outcome of transtrochanteric rotational osteotomy for osteonecrosis of the femoral head. J Bone Joint Surg Br..

[11] Zhao G, Yamamoto T, Ikemura S, Motomura G, Iwasaki K, Yamaguchi R (2012). Clinico-radiological factors affecting the joint space narrowing after transtrochanteric anterior rotational osteotomy for osteonecrosis of the femoral head. J Orthop Sci.

[12] Zhao G, Yamamoto T, Motomura G, Iwasaki K, Yamaguchi R, Ikemura S (2013). Radiological outcome analysis of transtrochanteric posterior rotational osteotomy for osteonecrosis of the femoral head at a mean follow-up of 11 years. J Orthop Sci.

[13] Sugioka Y, Hotokebuchi T, Tsutsui H (1992). Transtrochanteric anterior rotational osteotomy for idiopathic and steroid-induced necrosis of the femoral head: indications and long-term results. Clin Orthop Relate Res.

[14] Saito S, Ohzono K, Ono K (1988). Joint-preserving operations for idiopathic avascular necrosis of the femoral head. Results of core decompression, grafting and osteotomy. J Bone Joint Surg Br..

[15] Hasegawa Y, Iwata H, Mizuno M, Genda E, Sato S, Miura T (1992). The natural course of osteoarthritis of the hip due to subluxation or acetabular dysplasia. Arch Orthop Trauma Surg.

[16] Matsuo K, Hirohata T, Sugioka Y, Ikeda M, Fukuda A (1988). Influence of alcohol intake, cigarette smoking, and occupational status on idiopathic osteonecrosis of the femoral head. Clin Orthop Relate Res.

[17] Lamb KE, Thornton LE, Teychenne M, Milte C, Cerin E, Ball K (2017). Associations between access to alcohol outlets and alcohol intake and depressive symptoms in women from socioeconomically disadvantaged neighbourhoods in Australia. BMC Public Health.

[18] Sugano N, Atsumi T, Ohzono K, Kubo T, Hotokebuchi T, Takaoka K (2002). The 2001 revised criteria for diagnosis, classification, and staging of idiopathic osteonecrosis of the femoral head. J Orthop Sci.

[19] Yamamoto T, Ikemura S, Iwamoto Y, Sugioka Y (2010). The repair process of osteonecrosis after a transtrochanteric rotational osteotomy. Clin Orthop Relate Res.

[20] Ikemura S, Yamamoto T, Jingushi S, Nakashima Y, Mawatari T, Iwamoto Y (2007). Use of a screw and plate system for a transtrochanteric anterior rotational osteotomy for osteonecrosis of the femoral head. J Orthop Sci.

[21] Iqbal F, Younus S, Asmatullah ZOB, Khan N (2017). Surgical site infection following fixation of acetabular fractures. Hip Pelvis.

[22] Duckworth AD, Bennet SJ, Aderinto J, Keating JF (2011). Fixation of intracapsular fractures of the femoral neck in young patients. J Bone Joint Surg Br.

[23] Garcia-Sanchez A, Gonzalez-Calvin JL, Diez-Ruiz A, Casals JL, Gallego-Rojo F, Salvatierra D (1995). Effect of acute alcohol ingestion on mineral metabolism and osteoblastic function. Alcohol Alcohol.

[24] Chakkalakal DA, Novak JR, Fritz ED, Mollner TJ, McVicker DL, Lybarger DL (2002). Chronic ethanol consumption results in deficient bone repair in rats. Alcohol Alcohol.

[25] Janicke-Lorenz J, Lorenz R (1996). Alcoholism and fracture healing. A radiological study in the rat. Arch Orthop Trauma Surg.

[26] Chakkalakal DA, Novak JR, Fritz ED, Mollner TJ, McVicker DL, Garvin KL (2005). Inhibition of bone repair in a rat model for chronic and excessive alcohol consumption. Alcohol.

[27] Cordero-Ampuero J, Dios M (2010). What are the risk factors for infection in hemiarthroplasties and total hip arthroplasties?. Clin Orthop Relate Res.

[28] Klekamp J, Spengler DM, McNamara MJ, Haas DW (1999). Risk factors associated with methicillin-resistant staphylococcal wound infection after spinal surgery. J Spinal Disord.

[29] McPhee IB, Williams RP, Swanson CE (1998). Factors influencing wound healing after surgery for metastatic disease of the spine. Spine.

[30] Snäll J, Apajalahti S, Suominen AL, Törnwall J, Thorén H (2015). Influence of perioperative dexamethasone on delayed union in mandibular fractures: a clinical and radiological study. Med Oral Patol Oral Cir Bucal.

